# Label-Free CD34+ Cell Identification Using Deep Learning and Lens-Free Shadow Imaging Technology

**DOI:** 10.3390/bios13120993

**Published:** 2023-11-21

**Authors:** Minyoung Baik, Sanghoon Shin, Samir Kumar, Dongmin Seo, Inha Lee, Hyun Sik Jun, Ka-Won Kang, Byung Soo Kim, Myung-Hyun Nam, Sungkyu Seo

**Affiliations:** 1 Department of Electronics and Information Engineering, Korea University, Sejong 30019, Republic of Korea; als5dud@korea.ac.kr (M.B.); ghost10s@korea.ac.kr (S.S.); skumar@korea.ac.kr (S.K.); 2 Department of Electrical Engineering, Semyung University, Jecheon 27136, Republic of Korea; dseo@semyung.ac.kr; 3 Department of Biotechnology and Bioinformatics, Korea University, Sejong 30019, Republic of Korea; dlsgk1017@korea.ac.kr (I.L.); toddjun@korea.ac.kr (H.S.J.); 4 Department of Hematology, Anam Hospital, Korea University College of Medicine, Seoul 02841, Republic of Korea; ggm1018@korea.ac.kr (K.-W.K.); kbs0309@korea.ac.kr (B.S.K.); 5 Department of Laboratory Medicine, Anam Hospital, Korea University College of Medicine, Seoul 02841, Republic of Korea

**Keywords:** CD34+ classification, lens-free shadow imaging, Cellytics, artificial intelligence, deep learning, label-free cytometry, leukemia diagnosis, point-of-care diagnosis

## Abstract

Accurate and efficient classification and quantification of CD34+ cells are essential for the diagnosis and monitoring of leukemia. Current methods, such as flow cytometry, are complex, time-consuming, and require specialized expertise and equipment. This study proposes a novel approach for the label-free identification of CD34+ cells using a deep learning model and lens-free shadow imaging technology (LSIT). LSIT is a portable and user-friendly technique that eliminates the need for cell staining, enhances accessibility to nonexperts, and reduces the risk of sample degradation. The study involved three phases: sample preparation, dataset generation, and data analysis. Bone marrow and peripheral blood samples were collected from leukemia patients, and mononuclear cells were isolated using Ficoll density gradient centrifugation. The samples were then injected into a cell chip and analyzed using a proprietary LSIT-based device (Cellytics). A robust dataset was generated, and a custom AlexNet deep learning model was meticulously trained to distinguish CD34+ from non-CD34+ cells using the dataset. The model achieved a high accuracy in identifying CD34+ cells from 1929 bone marrow cell images, with training and validation accuracies of 97.3% and 96.2%, respectively. The customized AlexNet model outperformed the Vgg16 and ResNet50 models. It also demonstrated a strong correlation with the standard fluorescence-activated cell sorting (FACS) technique for quantifying CD34+ cells across 13 patient samples, yielding a coefficient of determination of 0.81. Bland–Altman analysis confirmed the model’s reliability, with a mean bias of −2.29 and 95% limits of agreement between 18.49 and −23.07. This deep-learning-powered LSIT offers a groundbreaking approach to detecting CD34+ cells without the need for cell staining, facilitating rapid CD34+ cell classification, even by individuals without prior expertise.

## 1. Introduction

Cell markers are proteins, glycosylations, or other biological components essential for the identification and classification of different cell types [1,2]. Interactions between cell surface epitopes and receptors or ligands can be used to confirm infection states or diagnose diseases by measuring the expression of specific markers [3]. Among these markers, the cluster of differentiation (CD) marker CD34 is employed to identify hematopoietic stem cells [4]. Stem cells in the bone marrow can develop into red blood cells, white blood cells, or platelets. CD34, a glycoprotein, is primarily expressed in hematopoietic stem cells, constituting 1–3% of normal bone marrow and 0.01–0.1% of white blood cells in the peripheral blood [5]. CD34 is expressed primarily in the earliest stages of hematopoietic stem cell development, and its expression decreases as the cells mature [6]. Detection of CD34+ cells, cells expressing CD34, is crucial for monitoring diseases such as leukemia and hematopoietic stem cell transplantation [4,7,8]. Dysregulated proliferation at any stage of hematopoietic cell differentiation can lead to the abnormal accumulation of CD34+ cells in the bone marrow or peripheral blood, making these markers particularly significant for the diagnosis of diseases such as leukemia, characterized by abnormal growth [9].

The CD34+ cell classification is important for the diagnosis of leukemia for several reasons. First, it serves as a valuable tool for distinguishing between various leukemia types, including acute myeloid leukemia (AML) and chronic myeloid leukemia (CML). Second, it provides critical insights into the prognosis of leukemia patients, with higher percentages of CD34+ cells in AML patients correlating with poorer outcomes than those with lower percentages [10]. Thirdly, CD34+ cell classification plays a pivotal role in monitoring the response of leukemia patients to treatment [11].

Leukemia is diagnosed when more than 20% of immature cells (blasts) in the bone marrow contain the CD34+ marker [12,13,14]. The percentage of CD34+ cells exhibits notable variation, ranging from 40 to 60% in AML and 70% or more in pediatric B-cell precursor acute lymphoblastic leukemia (ALL) to approximately 0–46% in T-cell acute lymphoblastic leukemia [10,15,16]. Moreover, CD34+ is a crucial indicator for determining the timing of hematopoietic stem cell collection for leukemia treatment [17,18,19,20,21]. Treatment strategies for patients with leukemia rely on multiple factors, including the disease subtype, overall patient health, and donor availability. Hematopoietic stem cells, sourced from either a donor or the patient’s peripheral blood or bone marrow, are harvested and subsequently transplanted. Given the low concentration of these cells in the source material, drugs such as granulocyte colony-stimulating factor (G-CSF or GM-CSF) are administered to stimulate their proliferation over 3 to 5 days [22,23]. The stem cells are then harvested via a central venous catheter or a large arm vein over a span of 2 to 3 days. Successful transplantation mandates a minimum count of 2 × 10^6^/kg hematopoietic stem cells [24]. Therefore, continuous cell counting using flow cytometry is performed to assess the suitability of the samples for transplantation. To accurately determine the number of hematopoietic stem cells, the number of cells expressing CD34+ must be assessed. As a result, continuous cell counting using flow cytometry is conducted to assess the suitability of the sample for transplantation. Therefore, the precise quantification of CD34+ cells is of the utmost importance for the diagnosis and treatment of leukemia.

Flow cytometry is the gold standard method for CD34+ cell measurement, which utilizes lasers to assess cell characteristics using scattering and fluorescent antibodies [25,26,27]. Typically, this technique involves the binding of specific fluorescent markers to cells to quantify the desired cell population [28,29]. Effective implementation of flow cytometry requires a comprehensive understanding of cell surface antigens, antibody responses, and fluorescent conjugate synthesis, all of which require specialized knowledge of advanced fluorescent staining techniques. To ensure sample reliability, the analysis is conducted on individual cells surpassing a predefined threshold, contributing to the time-intensive nature of the preparation and measurement processes. Flow cytometry is a complex technique requiring trained personnel and calibrated equipment. Qualified individuals are essential for supervising the various stages of the instrument’s operation, and discrepancies in the interpretation of results between different laboratories are often observed [30,31]. Furthermore, the flow cytometry instruments are bulky and expensive.

Various compact devices have been developed to overcome these limitations and offer innovative solutions. For example, NanoEntek introduced ADAM2, which uses a cell chip to measure CD34+ cells [32,33]. ADAM2 uses an imaging camera to count CD34+ cells and determine their concentrations in a given sample. This device has the advantages of reduced cost and enhanced accessibility to non-experts. Nevertheless, this method still relies on fluorescent staining for selective counting of CD34+ cells in a sample [34]. This staining approach has limitations, particularly when dealing with samples from medical facilities such as hospitals, which necessitates advanced knowledge of fluorescent staining and specific handling conditions to prevent extinction. These limitations contribute to the risk of sample deterioration over time. Therefore, there is a pressing need to develop a portable and user-friendly technique for quantifying CD34+ levels without the requirement for separate fluorescent staining, thus minimizing the risk of sample degradation.

Lens-free shadow imaging technology (LSIT) is a well-established method for characterizing microparticles and biological cells [35]. In LSIT, the diffraction pattern produced by an object is directly recorded using CCD/CMOS technology, thereby avoiding the use of lens elements and offering a broad field of view [36,37]. LSIT is known for its simplicity and cost-effectiveness, making it a popular choice for diverse low-cost applications, including the automated analysis of a complete blood count, cell morphology, microalgae toxicity, and cell viability, all without the need for staining [38,39,40,41,42,43].

In LSIT, cell properties are assessed by examining the diffraction pattern of light emitted from individual cells, eliminating the need for additional procedures such as staining for measurement. Images obtained using LSIT are analyzed using statistically derived parameters, such as the central maxima value (CMV), peak-to-peak distance (PPD), maxima-to-minima distance (MMD), and standard deviation of MMD (SMD) [36,44,45]. These parameters facilitate the analysis of various cellular attributes including cell viability, morphology, size, and type. Furthermore, there is an opportunity to enhance the accuracy and precision of LSIT using advanced techniques such as deep learning (DL), which has garnered considerable attention in recent years [35].

DL is a branch of machine learning (ML) and artificial intelligence (AI) that uses multiple layers of neural networks to learn from data [46,47,48]. DL plays a pivotal role in the Fourth Industrial Revolution and has applications across diverse domains including healthcare, visual recognition, natural language processing, and cybersecurity [49,50,51]. Many sophisticated models have emerged within the DL domain, including AlexNet artificial neural networks (ANN), convolutional neural networks (CNN), recurrent neural networks (RNN), and graph neural networks (GAN) [47,52]. These models are engineered to autonomously recognize complex patterns in various data types ranging from images to text and audio, effectively surmounting the limitations of traditional rule-based algorithms [53,54,55,56,57,58]. Consequently, the utilization of DL techniques for analyzing images obtained through LSIT has the potential to substantially enhance the existing statistical parameters and even create entirely novel methods for characterizing and assessing cell structures.

In this study, we analyzed the images of blood samples obtained by LSIT using a customized AlexNet DL model to selectively identify and quantify CD34+ cells in the samples, obviating the requirement for fluorescent staining. Blood samples were collected from the bone marrow of individuals with suspected or confirmed diagnosis of leukemia. Peripheral blood samples were collected from the selected patients. The mononuclear cells (MNC) obtained underwent a purity assessment via fluorescence-activated cell sorting (FACS) and were subsequently analyzed using our proprietary LSIT-based device, Cellytics. The cell images were categorized into CD34+ cells and other residual cells to generate a labeled training dataset. This dataset, comprising 10,000 training images, was used to train the custom AlexNet model to distinguish CD34+ cells from residual cells without staining. The performance of the DL model was assessed using fresh blood samples from 13 patients, with these same samples subjected to FACS analysis for validation. Our custom AlexNet model exhibited an impressive 97.32% accuracy in distinguishing CD34+ cells and demonstrated an average difference of 8.99% compared to FACS, with a coefficient of determination of *R*^2^ = 0.81. This DL-based approach eliminates the need for separate staining procedures, enabling the rapid and precise detection of CD34+ cells, thereby proving invaluable in leukemia diagnosis, hematopoietic stem cell transplant monitoring, and similar research applications.

## 2. Materials and Methods

### 2.1. Analysis Procedure

**Figure 1:**
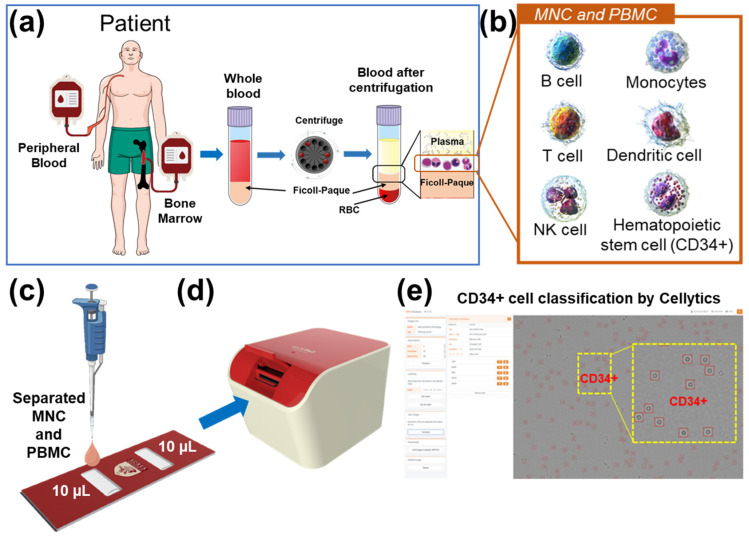
(**a**) Blood samples from the bone marrow and peripheral blood were collected and separated using Ficoll density gradient centrifugation. (**b**) Mononuclear cell samples were separated from various cell types, including blasts. (**c**) Ficoll-separated samples were injected directly into a cell chip. (**d**) The cell chip was inserted into the Cellytics device, which recorded shadow images of the cells. (**e**) Cellytics rapidly classifies only cells in samples using a CD34+ marker. The identified CD34+ cells within the red circle is illustrated in the magnified yellow box.

This study primarily focused on the detection of CD34+ markers within immature leukemia cells (blasts) present in blood samples acquired from the bone marrow and peripheral blood of patients. Figure 1 provides a comprehensive depiction of the sample preparation and workflow employed for CD34+ cell identification. The collected samples were separated to isolate mononuclear cells (MNC) and peripheral blood mononuclear cells (PBMC) by Ficoll density gradient centrifugation (Figure 1a). Subsequently, Ficoll-separated samples containing various cell types, including lymphocytes, monocytes, granulocytes, and blasts, were directly introduced into a cell chip without any additional treatment (Figure 1b,c). The cell chip, constructed from optically transparent polycarbonate, featured two chambers, each capable of holding 10 μL of the sample. The cell chip was then inserted into the Cellytics device, which captured diffraction images of the cells in the sample (Figure 1d). Cellytics can identify and distinguish CD34+ cells from shadow images of samples with diverse cell types (Figure 1e). This selective identification allowed for the determination of the CD34+ cell percentage in the sample.

**Figure 2:**
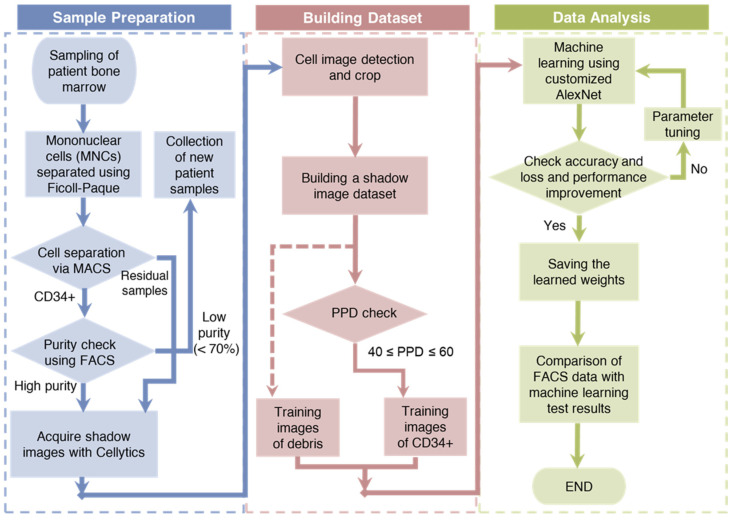
A schematic representation of the research process, divided into three main phases: sample preparation, dataset generation, and data analysis.

The research process was divided into three main phases: sample preparation, dataset generation, and data analysis (Figure 2). During the initial sample preparation phase, bone marrow samples were sourced from hospitalized patients and served as the primary material for this study. These samples were meticulously separated into mononuclear cells (MNCs) using Ficoll density gradient centrifugation. The isolated MNCs were subsequently subjected to magnetic-activated cell sorting (MACS), which effectively classifies CD34+ and residual CD34 cells according to the established MACS protocol. The purity of the CD34+ cells was rigorously checked using FACS, ensuring a minimum purity of 70%. Samples with purity above this threshold were further processed using Cellytics to obtain a comprehensive collection of CD34+ cell images. In contrast, the remaining cells were directly processed with Cellytics without the requirement for separate purity confirmation, ultimately yielding extensive compilation of CD34 cell images.

The second phase, the generation of a robust dataset from the acquired CD34+/CD34 cell images, involved several crucial steps. First, background noise was efficiently removed by computing the average pixel values across the entire image. Next, the remaining pixels were meticulously scrutinized to pinpoint individual cell centers, clearly demarcated with red boxes measuring 30 × 30 pixels each. These regions were then cropped to form the fundamental training dataset, thereby ensuring a consistent image size for the training data. However, because of the origin of CD34+ training data from samples with a minimum purity of 70%, a selection process was necessary. For this selection, the PPD parameter was employed, with only the training data falling within the PPD range of 40 to 60, which was categorized as CD34+. In this study, a total of 10,000 CD34+ and residual cell training images were compiled [59].

In the final phase, a customized version of the AlexNet architecture called customized AlexNet was used as the DL model. The hyperparameters of this model were systematically fine-tuned, and the validation accuracy and loss were closely monitored, leading to the derivation of optimized weights. Using these optimized weights, the percentage of CD34+ cells in patient samples was assessed and the results were compared with FACS measurements, serving as the performance benchmark for comparison.

### 2.2. Sample Preparation

This study was approved by the Institutional Review Board (IRB:2021AN0040) of the Anam Hospital of Korea University. A total of 18 outpatients with suspected or diagnosed leukemia contributed to this research. Bone marrow and peripheral blood samples were collected and stored in anticoagulant tubes at 4 °C until utilization. Five patients, meeting a minimum CD34+ purity threshold of 70%, were the source of training data for the customized AlexNet model, whereas the remaining 13 blinded samples (comprising 10 bone marrow samples and 3 peripheral blood samples) were employed to validate the model’s performance. The buffer solution used for the blood experiments consisted of Dulbecco’s phosphate-buffered saline (DPBS; Merck KGaA, Darmstadt, Germany) supplemented with 0.5% bovine serum albumin (BSA; Bovogen Biologicals, Keilor East, VIC, Australia) and 2 mM ethylenediaminetetraacetic acid (EDTA; LPS Solution, Daejeon, Republic of Korea).

### 2.3. Separation of CD34+ to Build Training Data 

#### 2.3.1. Extraction of MNCs from Bone Marrow Blood

To isolate mononuclear cells (MNCs) from the obtained bone marrow aspirates, a density gradient centrifugation method employing Ficoll-Paque was used. The samples were initially diluted in a 1:1 ratio with phosphate-buffered saline (PBS). Subsequently, these diluted samples were meticulously layered on Ficoll-PaqueTM Premium 1.084 medium (GE Healthcare Life Sciences, Uppsala, Sweden) and centrifuged at 445× *g* for 30 min at 25 °C. Following centrifugation, the MNC layer was carefully collected in a 15 mL conical tube. To cleanse the collected MNCs, 10 mL of PBS was introduced, and the cells were gently mixed and centrifugated once more at 400× *g* for 10 min at 25 °C, ensuring thorough removal of the supernatant.

#### 2.3.2. Isolation of CD34+ Cells from MNCs

In this study, MACS was used to isolate CD34+ cells from the MNCs. MNCs were labeled with CD34 magnetic beads (Miltenyi Biotec, Bergisch Gladbach, Germany). The labeled sample was introduced into a magnetic column, where cells other than CD34+ cells traversed the column and were subsequently extracted. The magnetic field was then disengaged from the column to collect CD34+ cells. The protocol used for this procedure was performed according to the manufacturer’s instructions [60].

#### 2.3.3. Purity Check of Extracted CD34+ Cells

The purity of the CD34+ cells derived from the bone marrow was evaluated using flow cytometry. CD34+ cells were conjugated with anti-human CD34-FITC (Miltenyi Bio-tec, Bergisch Gladbach, Germany), and FACSCantoⅡ^TM^ (Becton Dickinson, CA, USA) was used for the analysis. This process was performed according to the manufacturer’s guidelines [61]. The purity of isolated CD34+ cells was at least 70%.

### 2.4. Building the Training Dataset

#### 2.4.1. Cell Image Acquisition

To create a training dataset, an array of cell images was acquired using the Cellytics system. Cellytics has dimensions of 100 × 120 × 80 mm^3^ and weighs 550 g. It utilizes a 470 ± 5 nm blue LED (LB W5SM-FZHX-35, Osram, Munich, Germany) as the point light source and incorporates a 300 μm pinhole for the generation of semi-coherent light. The interaction of this semi-coherent light with the sample produces diffraction patterns arising from the interference between the light traversing the sample and light refracted within the sample. The resulting diffraction patterns were captured using a CMOS image sensor (MT9P031I12STM- DP, ON Semiconductor, Phoenix, AZ, USA) and stored as images with dimensions of 2592 × 1944 pixels. The captured cell images were subsequently divided into two classes: CD34+ and CD34^−^.

#### 2.4.2. Building the Dataset 

To construct the dataset for deep learning analysis, individual cell images of a standardized size were generated from a substantial collection of cell images captured by Cellytics facilitated by an object recognition algorithm. The algorithm first removed the background by averaging the pixel values of the entire image. Subsequently, pixel by pixel, the entire image was scrutinized for pixels with a value of 1. Upon identifying such pixels, the algorithm detected adjacent pixels and altered the intermediate pixels to a value of 1 if the neighboring pixels also possessed a value of 1. The algorithm then calculated the center of the modified image and generated a red rectangle measuring 30 × 30 pixels around the center. The data located within this created rectangle in the original image were cropped and saved as a single object, serving as training data. An image transformed to a value of 1 was recognized as a cell if its width and height fell within the range of 8 to 40 pixels or beyond. The object recognition algorithm was instrumental in producing single-cell images, each sized at 30 × 30 pixels, which were subsequently employed as training data.

### 2.5. Cell Analysis 

The DL training and performance analysis in this study were conducted using the PyTorch library in Python. The hardware for the experiment execution was a Dell Inspiron 15 Gaming i15-7567 notebook, 8 GB of 2133 MHz DDR4 RAM Memory, a 7th Generation Intel Core i5-7300HQ Quad Core processor (with 6MB Cache, up to 3.5 GHz), a 1 TB 5400 rpm HD with 8 GB cache, and an NVIDIA GeForce RTX 3080ti GPU (Dell, Round Rock, TX, USA).

The deep learning model used in this study was AlexNet, which is a well-established CNN model. Our customized AlexNet featured eight convolutional layers, five max-pooling layers, and three fully linked layers. The activation function employed was the scaled exponential linear unit (SELU), represented mathematically as

(1) $$\mathrm{SELU}\;\left(x\right)=\lambda \{\begin{array}{rr} \;\alpha e^{x}-\alpha & \mathrm{if}\;x<0\\ x & \mathrm{if}\;x\geq 0 \end{array}$$

**Figure 3:**
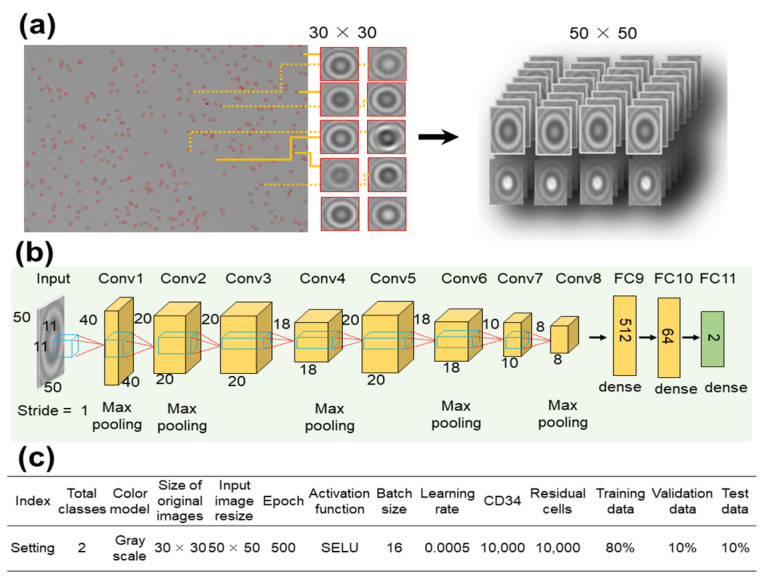
Architecture of the customized AlexNet deep learning model for CD34+ cell classification. (**a**) Input cell images of size 30 × 30 pixels were resized to 50 × 50 pixels and (**b**) went through eight convolutional layers. (**c**) Hyperparameters used to train the modified customized AlexNet model.

Here, the SELU parameters were set to the commonly used values of *λ* = 1.0507 and *α* = 1.6732. Figure 3 shows the customized AlexNet model employed in this study. We selected AlexNet because of its simplicity and capability to capture spatial features relevant to cell-shape analysis. 

The 30 × 30 pixels object images were resized to 50 × 50 pixels (as shown in Figure 3a) and processed through eight convolutional layers. During this process, resizing was accomplished through max pooling in Conv1, Conv2, Conv4, Conv6, and Conv8 (Figure 3b). Finally, the images were passed through three fully connected (FC) layers to complete the learning process. A summary of the settings employed to train the customized AlexNet model is shown in Figure 3c.

The dataset, consisting of 20,000 images, was randomly divided into training, validation, and test groups at an 8:1:1 ratio. The images were labeled as either CD34+ or residual cells. The network underwent training for 500 epochs with a batch size of 16 and a learning rate of 0.0005. The performance of the model was compared to that of two other pre-trained CNN models: Visual Geometry Group 16 (VGG16) and ResNet50.

### 2.6. Performance Verification 

To evaluate the performance of our DL model for CD34+ cell analysis, bone marrow samples from 10 patients and peripheral samples from 3 patients were used. These blinded validation samples underwent processing to isolate mononuclear cells (MNCs) and peripheral blood mononuclear cells (PBMCs). Subsequently, these samples were subjected to analysis using FACS, the current gold standard technique, and our customized AlexNet model.

FACS involves fluorescently staining the samples and analyzing approximately 10,000 cells to determine the percentage of CD34+ cells among the measured cells. In contrast, our custom AlexNet model analyzed unlabeled cell samples with a concentration of approximately 10^5^ cells, all without the need for fluorescent staining, to ascertain the CD34+ percentage. The results obtained from these two methods were then compared using regression analysis to compute the coefficient of determination (R-squared value) and were visualized with a Bland–Altman plot.

## 3. Results and Discussion

The samples used to generate individual cell images had a CD34+ cell purity of 70%. However, to account for potential errors stemming from the presence of 30% residual cells, a data selection process was implemented. Gradient-weighted class activation mapping (Grad-CAM) was applied for localizing the CD34+ cells within the images. Grad-CAM is an algorithm that visualizes the regions considered important for discriminating CD34+ images from residual cell images, providing insight into the crucial areas within each image [62,63].

**Figure 4:**
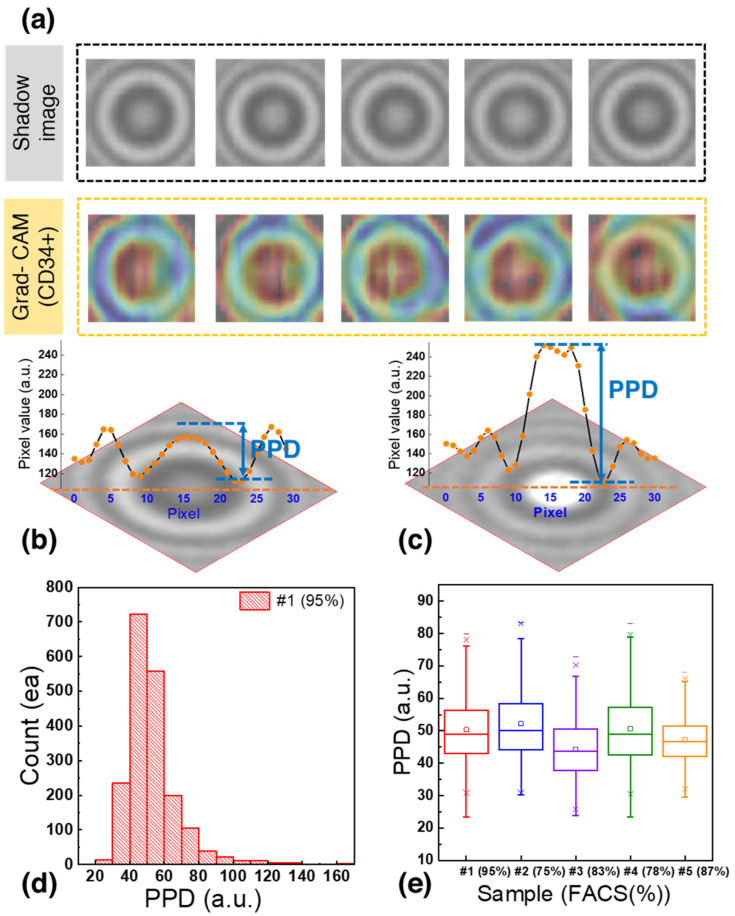
(**a**) Shadow image and corresponding gradient-weighted class activation mapping (Grad-CAM) heatmap of CD34+ cells. (**b**) Shadow image of a CD34+ cell with a PPD of 44.3. (**c**) Shadow image of a residual cell with a PPD of 96.3. (**d**) PPD distribution of 1929 bone marrow cells used to generate the training dataset. (**e**) Boxplot of PPD distribution of CD34+ cells.

In Figure 4a, the region between the bright central point and the first dark ring encircling the center of the image is highlighted in red. Analysis of pixel values in this area could allow the classification of images as CD34+ or CD34^–^− Previous studies have introduced statistical parameters for the analysis of diffraction patterns [38]. In this study, the parameter for the highlighted area was designated as PPD. PPD quantifies the difference between the pixel value at the center of the image and the lowest value of the first dark concentric ring, and it is closely related to the size and shape of the cells [44].

Figure 4b,c shows representative shadow images of CD34+ cells and residual cells, respectively, with their corresponding pixel values. The CD34+ cell (Figure 4b) had a central pixel value of 156.8 and the lowest pixel value of the dark concentric ring was 112.5, resulting in a PPD of 44.3. In contrast, the residual cells (Figure 4c) had a PPD of 96.3, representing a difference of 52.0 compared to the CD34+ cells.

Figure 4d shows the PPD distribution for 1929 bone marrow cells analyzed to generate the training dataset. Notably, 95% of the cells fell within the PPD range of 20 to 80 with 66.3% of them falling within the PPD range of 40 to 60. This distribution provides valuable insights into the characteristics of the CD34+ and residual cells in the dataset.

Figure 4e shows a boxplot based on the data from Figure 4d. After calculating the interquartile range (IQR) and removing outliers, it was found that cells with the CD34+ marker exhibited a PPD distribution within the range of 43.5 to 58, encompassing 50% of the distribution. To further confirm the PPD range of CD34+ cells, blood samples from the bone marrow of four patients were analyzed. It was observed that 50% of the cells in these samples fell within the following PPD ranges: 44 < PPD < 58, 37.84 < PPD < 52.25, 43 < PPD < 58.5, and 42.6 < PPD < 53.5. Since the PPD ranges overlapped across multiple patient samples, images with PPD values between 40 and 60 were selected and labeled as CD34+ to train the deep learning model. This approach aimed to enhance the accuracy of identifying cells expressing the CD34+ marker while accommodating the variability between patient samples and ensuring the creation of a robust training dataset for classification.

**Figure 5:**
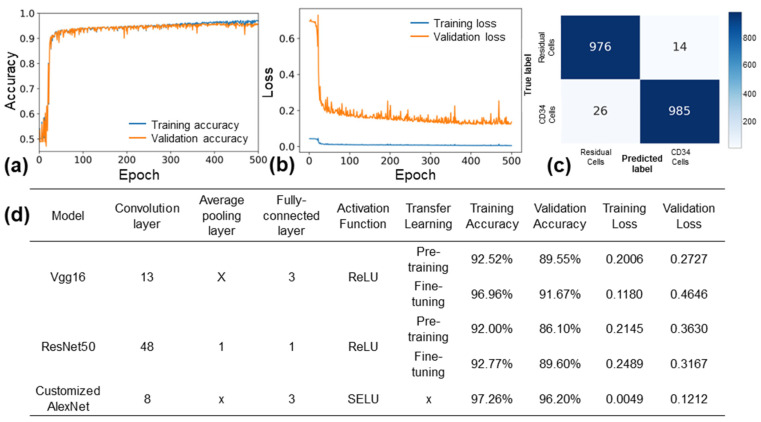
Performance of customized AlexNet model. (**a**) Training and validation accuracy, and (**b**) training and validation loss of customized AlexNet model with optimized hyperparameters. (**c**) Confusion matrix of the model for a test set of 2000 images. (**d**) Comparison of the customized AlexNet model with the VGG16 and ResNet50 models.

The hyperparameters of the custom AlexNet model were systematically optimized by varying the batch size (8, 16, 32), learning rate (0.0001, 0.0005, 0.001), and the number of epochs (300, 500, 800, 100). The most favorable results were achieved with a batch size of 16, a learning rate of 0.0005, and an epoch number of 500. With these hyperparameters, the custom AlexNet model demonstrated training and validation accuracies of 97.3% and 96.2%, respectively, while maintaining a loss of 0.121 (refer to Figure 5a,b). 

In Figure 5c, the evaluation results of the custom AlexNet model on a test set comprising 2000 data points are presented. The confusion matrix reveals a precision of 99.2% and a recall of 98.4%. Figure 5d displays the outcomes of training the custom AlexNet, Vgg16, and ResNet50 models using the same images over 500 epochs. Notably, Vgg16 (fine-tuning) achieved a validation accuracy of 91.67% with a loss of 0.46, while ResNet50 (fine-tuning) recorded a validation accuracy of 89.6% and a loss of 0.31. These accuracy and loss results highlight the superior performance of the custom AlexNet model over Vgg16 and ResNet50. The custom AlexNet’s enhanced performance can be attributed to its simpler architecture, better suited for the relatively small and monochrome diffraction pattern images employed in this study. Additionally, the utilization of the SELU activation function, as opposed to the ReLU activation function, may have contributed to the model’s improved performance on monochrome images. SELU enables weights to be derived as nonzero values rather than negative values, potentially enhancing the model’s ability to learn from these images. Overall, the custom AlexNet model demonstrated outstanding performance in classifying CD34+ and residual cells from diffraction pattern images. 

**Figure 6:**
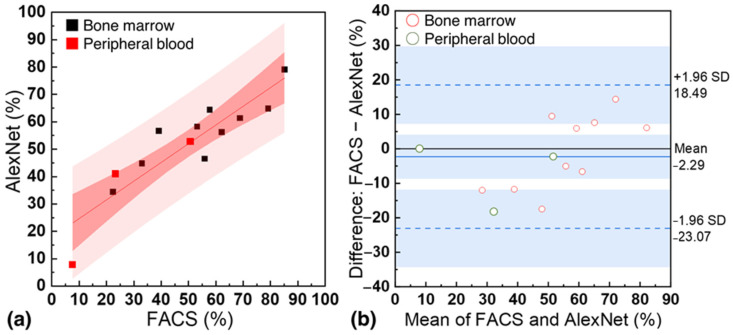
(**a**) Correlation between the percentage of CD34+ cells measured by standard flow cytometry (FACS) and the customized AlexNet model using samples from 13 patients. (**b**) Bland−Altman for FACS and the customized AlexNet model.

In Figure 6a, the performance comparison between FACS and the customized AlexNet model is presented using blood samples from 13 patients. The x-axis represents the percentage of CD34+ cells measured by FACS, while the y-axis represents the percentage of CD34+ cells measured by the customized AlexNet. CD34+ percentages of 22.4 to 85.2% (bone marrow) and 7.7 to 50.5% (peripheral blood) were measured with FACS, while 34.4 to 79.1% (bone marrow) and 7.65 to 52.83% (peripheral blood) were measured with the customized AlexNet based on a concentration of 10^5^ cells. The coefficient of determination (R2) between the two was 0.81, with an average F-difference of 8.99%. This demonstrates the strong agreement between the customized AlexNet model and FACS in quantifying CD34+ cells, highlighting the model’s accuracy and potential for clinical applications. Figure 6b presents a Bland−Altman plot with a base bias of −2.29 and 95% limits of agreement of 18.49 and −23.07. This further underscores the strong agreement between the standard FACS technique and the customized AlexNet model in quantifying CD34+ cells in patient samples, highlighting the model’s reliability and potential for clinical use.

The findings of this study present a significant advancement in leukemia diagnosis, specifically in the context of CD34+ cell classification, with several noteworthy implications:

1. Compact, low-cost LSIT-based device: The Cellytics device, in conjunction with the customized deep learning model, offers a cost-effective and portable solution for CD34+ cell detection. This compact and user-friendly device eliminates the need for complex sample preparation and dedicated facilities, making it suitable for point-of-care applications in clinical settings and resource-limited environments.
2. Enhanced accuracy and efficiency: The customized deep learning model demonstrates high accuracy in classifying CD34+ cells, outperforming other models. The validation of its performance in patient samples indicates its potential to improve the precision of leukemia diagnoses, allowing healthcare professionals to make informed decisions more reliably.
3. Streamlined diagnostic process: By eliminating the need for labor-intensive and expertise-dependent techniques, such as flow cytometry, this approach simplifies the diagnostic process. The combination of the Cellytics device and deep learning model may reduce the time and resources required for diagnosis.
4. Potential for clinical applications: Beyond leukemia diagnosis, this technology holds promise for various clinical applications, including hematopoietic stem cell transplantation monitoring. Accurate quantification of CD34+ cells can inform treatment decisions and enhance patient outcomes. To realize its potential, integration into clinical workflows would necessitate validation, standardization, regulatory approval, and data integration.

While this study has achieved significant progress in CD34+ cell classification for leukemia diagnosis, there are several limitations and avenues for future research to consider. Our model exhibited high accuracy; nonetheless, it is important to acknowledge that no diagnostic test is infallible, and false positives or negatives may still occur. This study included a relatively small number of patients, and further validation using larger and diverse patient cohorts across different populations and disease conditions is necessary to establish the robustness and generalizability of the approach. Furthermore, our study primarily focuses on identifying CD34+ cells in the context of leukemia. We recognize the importance of considering additional markers, such as CD33 and CD38, for a comprehensive characterization of leukemic blasts. In future research, we aim to expand our methodology to encompass a broader range of markers to provide a more complete phenotypic profile of haematopoietic cells, which would improve its clinical utility for leukemia characterization. In comparison to traditional methods like flow cytometry, the proposed approach offers several advantages as discussed above. However, it is important to note that flow cytometry remains the gold standard method for CD34+ cell quantification, and further validation and comparison studies are needed to establish the full utility and reliability of the proposed approach. Additional work is also needed to integrate automated sample loading and analysis capabilities into the Cellytics device to enable true sample-to-result functionality suitable for nonexpert use. 

## 4. Conclusions

In this study, we developed a customized AlexNet deep learning model designed for the classification of CD34+ and residual cells from shadow images obtained through an LSIT-based device known as Cellytics. The use of LSIT eliminates the necessity for cell staining, resulting in significantly reduced analysis time and resource requirements (approximately 15 min). The model exhibited exceptional performance when trained on a dataset comprising 20,000 bone marrow cell images, achieving a training/validation accuracy of 97.3/96.2% with a loss of 0.121. Furthermore, our customized AlexNet outperformed established models such as VGG16 and ResNet50. Validation using samples from 13 leukemia patients demonstrated a high level of agreement with flow cytometry in quantifying CD34+ cells. This innovative technique allows for marker detection without the need for staining, offering several key advantages over traditional methods. The customized AlexNet’s ability to analyze images with enhanced accuracy and efficiency contributes to reduced diagnostic time and resource utilization. It also exhibits reduced dependency on human expertise, resulting in improved reproducibility and reliability. Additionally, the model can handle images from diverse sample sources, including bone marrow and blood. In summary, the customized AlexNet represents a promising tool for the classification of CD34+ cells from diffraction images acquired using Cellytics (MetaImmuneTech Inc., Sejong, Korea). Future development efforts will aim to expand the dataset, conduct validation studies, optimize the model’s integration into clinical practice, apply this approach to various cell types and diseases, and explore its integration with diagnostic tools like flow cytometry. This study underlines the feasibility of a machine-learning-based solution for quantitative cell analysis using label-free diffraction imaging.

## Data Availability

The data presented in this study are openly available in GitHub (San Francisco, CA, USA) at 10.5281/zenodo.10143543 reference number [59].
